# Acute Effects of Commercial Yerba Mate Products on Cardiometabolic Responses during Submaximal Cycling: Brewed to Perform?

**DOI:** 10.1016/j.cdnut.2026.107637

**Published:** 2026-01-16

**Authors:** Sarah Zaki, Rédina Berkachy, Razan Zein Eddine, Mona Zeidan, Omar Obeid, Imad Toufeili, Elie-Jacques Fares

**Affiliations:** 1 Department of Nutrition and Food Sciences, Faculty of Agricultural and Food Sciences, American University of Beirut, Riad El-Solh, Beirut, Lebanon; 2 School of Engineering and Architecture of Fribourg (HEIA-FR), University of Applied Sciences of Western Switzerland (HES-SO), Fribourg, Switzerland; 3 Public Integrity Research Group, Institute of Communication and Public Policy, Faculty of Communication, Culture and Society, Università della Svizzera italiana (USI), Lugano, Switzerland; 4 Department of Medicine, Faculty of Medicine and Health Sciences, University of Sherbrooke, Quebec, Canada

**Keywords:** Yerba mate, substrate oxidation, thermogenesis, metabolic parameters, obesity

## Abstract

**Background:**

Yerba mate (*Ilex paraguariensis*) is an herb native to South America known for its caffeine and polyphenol content, with suggested benefits for fat oxidation and energy metabolism. However, few studies have evaluated its brewed forms during exercise, particularly across different commercial brands.

**Objectives:**

This study aimed to evaluate and compare the acute effects of two commercially available brands of brewed yerba mate on substrate oxidation and energy expenditure in healthy and overweight/obese adults during very low- to moderate-intensity exercise.

**Methods:**

A randomized crossover study comprising 29 healthy adults (15 men and 14 women) consumed 4 different drinks across separate visits: 2 commercially brewed yerba mate brands (AYM and KYM), water, and water with 135 mg caffeine. Following each drink, participants underwent graded cycling exercise while energy expenditure (EE), respiratory exchange ratio (RER), heart rate (HR), and delta efficiency (DE) were measured using indirect calorimetry.

**Results:**

Both yerba mate brands had similar caffeine concentrations but different phenolic content, yet neither significantly affected EE, substrate oxidation, HR, or DE during exercise compared to control drinks. Sex-based differences emerged, with women exhibiting higher RER and HR at similar workloads, but no drink × sex interactions were found. Obesity status influenced HR, EE, and DE, with obese participants showing altered physiological responses. A significant 3-way interaction (drink × intensity × sex) was observed for RER (*P* = 0.013), particularly in men. No drink × obesity interactions were detected.

**Conclusions:**

Acute ingestion of brewed yerba mate, regardless of brand, did not enhance metabolic or cardiovascular responses during low- to moderate-intensity cycling. Body composition, particularly obesity status, had a more consistent influence on exercise efficiency than the short-term consumption of functional beverages like yerba mate.

## Introduction

Functional foods have gained attention for their health benefits and potential role in weight management. Dietary phytochemicals, including a range of polyphenols (isoflavones, flavonoids, catechins, and stilbenes) and alkaloids (capsaicin and caffeine), exhibit thermogenic properties by enhancing energy expenditure (EE), thereby increasing the body’s metabolic rate [[1], [2], [3]].

Tea, derived from the *Camellia sinensis* plant, is globally renowned for its health benefits, primarily attributed to its high polyphenol content, particularly catechins and their oxidation products, flavonols, and phenolic acids [4]. Regular consumption has been linked to reduced risks of cardiovascular diseases, certain cancers, and neurodegenerative disorders, largely due to its antioxidant and anti-inflammatory properties [5].

Yerba Mate (YM) (*Ilex paraguariensis*) is cultivated in South America, mainly in Brazil, Paraguay, and Argentina [6,7], and its consumption has spread to the United States and the Middle Eastern countries, including Lebanon and Syria [8,9]. Its consumption dates back many centuries, but its effects on health have only been studied recently.

YM is a rich source of bioactive compounds, including polyphenols, caffeine, and saponins [10,11]. These compounds are thought to be responsible for the physiological benefits of YM, particularly through their effects on antioxidant defense, inflammation, lipid metabolism, glucose regulation, and energy metabolism [12,13]. Polyphenol concentrations in YM have been demonstrated to be comparable with those in red wine and higher than those in green tea [14,15]. YM polyphenols have been shown to reduce oxidative stress and inflammation by scavenging reactive oxygen species and regulating inflammatory signaling pathways [10], whereas caffeine may increase sympathetic activation, promote lipolysis, and enhance fatty acid availability during rest and exercise [16]. In addition, saponins present in YM have been associated with hypocholesterolemic effects through reduced intestinal lipid absorption and modulation of bile acid metabolism [12,13]. This high phenolic content contributes to its remarkable antioxidant, anti-inflammatory, hypocholesterolemic, and hepatoprotective properties [6,7,17]. Branco et al. [18] linked the anticonvulsant and neuroprotective effects of YM to its high caffeine and phenolic content. Furthermore, its consumption may protect against cardiovascular disease and obesity [13,19]. The thermogenic effect of certain compounds in the YM has triggered research on their potential role in weight loss [[20], [21], [22]]. Acute ingestion of caffeine- and polyphenol-rich beverages such as YM can elicit rapid metabolic effects through well-described sympathoadrenal mechanisms. Caffeine inhibits phosphodiesterases, whereas plant polyphenols can inhibit catechol-O-methyltransferase, together amplifying and prolonging the action of noradrenaline on β-adrenergic receptors. This interaction increases intracellular cAMP and stimulates thermogenesis, leading within minutes (∼40 min) [23] to measurable rises in EE, shifts toward greater fat oxidation (lower RER), and modest increases in HR [24,25].

In addition, Martinet et al. [26] tested the thermogenic effect of YM and various other plants using indirect calorimetry. YM was demonstrated to have a positive effect on endurance and performance during moderate-intensity exercise. YM was the only drink to significantly decrease the respiratory coefficient, indicating an increase in fat oxidation compared with placebo [27], similar to other functional foods (i.e., green tea extract), which demonstrated similar outcomes even without exercise [3]. Alkhatib [28] found that 1 g of YM increased fat oxidation by 24% and decreased carbohydrate oxidation during exercise at an intensity range of 40% to 70% of peak oxygen uptake (VO2peak), corresponding to moderate exercise intensity. This benefit potentially applies to a large part of the population, especially as moderate-intensity exercise is adopted for weight loss, disease prevention, and performance endurance. The reported reduction in body fat mass and waist-hip ratio following YM supplementation suggests that YM may be a potential antiobesity agent [29].

Despite these promising findings, existing studies often rely on encapsulated forms of YM or isolated extracts, which do not reflect real-world consumption practices. Furthermore, few studies have compared the metabolic impact of different commercial brands of YM [[30], [31], [32]], and research in Middle Eastern populations remains scarce.

This study aims to evaluate and compare the effects of 2 commercially available brands of brewed YM on substrate oxidation and EE in healthy and overweight/obese adults during very low- to moderate-intensity exercise. Because YM naturally contains caffeine, and caffeine’s physiological effects are dose-dependent relative to body weight, a fixed serving may deliver different effective doses across individuals [16,33]. This provides a biologically plausible reason to examine whether BMI influences the acute metabolic response to YM. By simulating real-life consumption methods (i.e., brewed tea rather than capsules), this study seeks to provide ecologically valid insights into the potential role of YM in metabolic health and performance.

## Methods

### Study design

**FIGURE 1:**
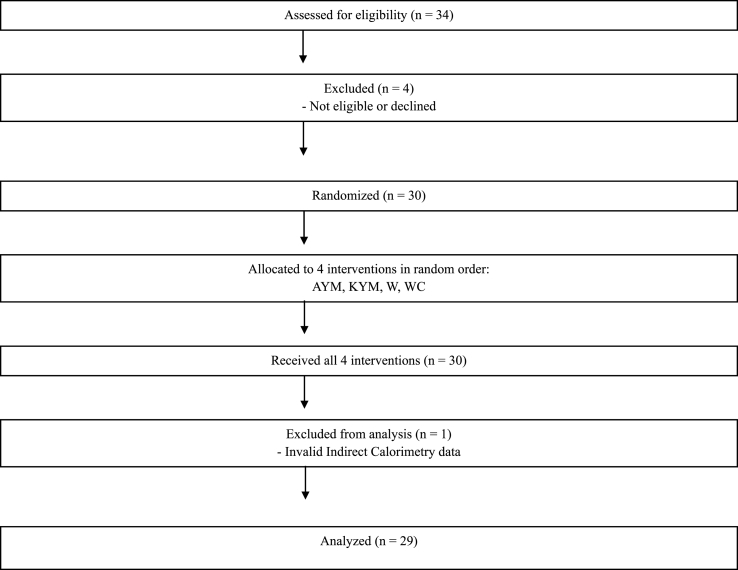
CONSORT Flow Diagram for a randomized crossover study in university students. Participants received 4 drinks on 4 occasions (YM drink—Amanda brand, AYM; YM drink—Kharta brand, KYM), water (W), and water + 135 mg of caffeine (WC). YM, yerba mate.

This study employed a randomized crossover design with 4 treatments as shown in Figure 1.

Figure 1 presents the CONSORT flow diagram outlining participant progression throughout the randomized crossover trial. Of the 34 individuals screened for eligibility, 30 met the inclusion criteria and were randomly assigned to receive the 4 study interventions: Amanda YM (AYM), Kharta YM (KYM), water (W), and water with 135 mg of caffeine (WC), in a counterbalanced order. All participants completed the 4 testing sessions. One participant was later excluded from the final analysis due to invalid indirect calorimetry measurements, resulting in a final analytical sample of 29 participants.

### Participants

Twenty nine participants (14 women and 15 men) aged 18 to 35 y, with a BMI of 24.4 ± 6.1 kg/m,^2^ were characterized by low caffeine (<200 mg/d) and tea (<2 tea bags/d or <4 g loose tea/d) consumption, nonsmoking status, and moderate or no alcohol intake (i.e., <14 standard drinks/wk for women and <21 standard drinks/wk for men). Subjects with physical limitations, cardiovascular disease, pregnancy, unstable body weight during the past 6 mo, or active weight loss were excluded from the study. Female participants were tested during the follicular phase of the ovarian cycle, determined based on self-reported menstrual cycle information (approximately days 4 to 11) [34,35]. This study was approved by the Institutional Review Board of the American University of Beirut (BIO-2022-0200). Informed consent was obtained from all participants. The participants were instructed to refrain from alcoholic and caffeinated drinks and to stop intense physical activity 48 h before the experiment. A minimum washout period of 3 d was maintained between experimental conditions.

### Procedures

#### Anthropometrics and body composition

All participants completed anthropometric measurements. Height was measured to the nearest 0.1 cm using a wall-mounted Seca stadiometer. Weight was assessed to the nearest 0.1 kg using a standard calibrated balance (Seca 869 Digital Floor Scale). Body composition parameters (i.e., fat and fat-free mass) were assessed using a bioelectrical impedance analysis device (InBody 770, InBody Co., Ltd.). Participants were asked to remove any clothing that might affect the measurement (i.e., heavy clothing and jewelry) and to stand barefoot on the foot electrodes. They were also asked to grip the hand electrodes to ensure proper contact with the device, stand upright with their arms slightly away from their body, and avoid any movement during the measurement.

#### Intervention

**FIGURE 2:**
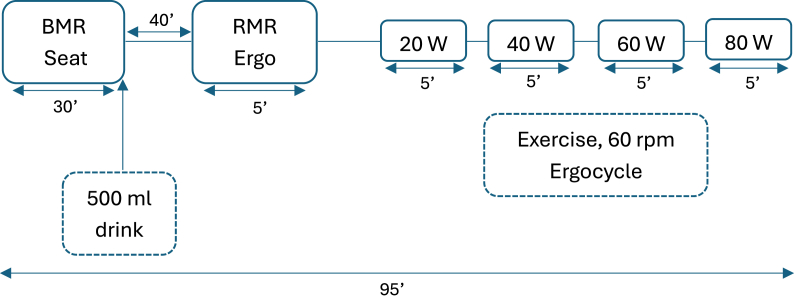
Intervention. BMR, basal metabolic rate; RMR, resting metabolic rate; Ergo, cycle ergometer; rpm, revolutions per min.

All participants visited the Nutrition and Metabolic Laboratory on 4 separate occasions, after 12 h overnight fast. At each visit, the basal metabolic rate (BMR) was measured for 30 min. YM infusions were prepared using a fully reproducible protocol. Specifically, 7.5 g of dried leaves were weighed on a calibrated laboratory scale and added to 500 mL of recently boiled water. The water was allowed to cool until it reached 90°C, as verified with a laboratory thermometer, before being poured over the leaves. The mixture was then left to brew undisturbed for 15 min and subsequently filtered through a fine mesh stainless-steel sieve to remove all plant material. All infusions were prepared fresh on the day of testing using the same procedure across sessions. After BMR assessment, participants were randomly given 500 mL of one of the following drinks: *1*) water, *2*) Amanda YM drink (7.5 g), *3*) Kharta Khadra YM drink (7.5 g), or *4*) water containing 135 mg caffeine. These YM brands were selected based on their widespread availability and high consumer demand in Lebanese supermarkets. Based on previous research, the minimum effective caffeine dose required to stimulate thermogenesis and influence fat metabolism is slightly >100 mg [30,31]. Following drink ingestion, participants rested for 40 min, after which resting metabolic rate (RMR) was measured. Then, the participants started pedaling on an ergometer (E100, ergo, COSMED) at 60 revolutions per minute. The initial intensity was 20 Watts (W), with an increase of 20 W every 5 minutes (20, 40, 60, and 80 W). This range of power was chosen to simulate real-life low- to moderate- intensities (1.4–6 METs) [36,37]. The total pedaling duration was 20 minutes (Figure 2).

During the measurements (BMR, RMR, and dynamic exercise), participants were asked to wear a silicone facemask connected to a Quark Cardio Pulmonary Exercise Testing (CPET) indirect calorimeter (COSMED) to analyze the respiratory parameters. Prior to each test, Quark CPET was calibrated according to the manufacturer’s instructions. Handlebar and saddle heights of the ergometer were adjusted for each participant.

### YM content

#### Total phenolic content

The total phenolic content of the brews was determined using the Folin-Ciocalteu reagent according to Singleton et al. [38]. An aliquot of the mate brew (200 μL) was mixed with 1mL of diluted Folin-Ciocalteu reagent (1:10, v/v; in water). Four minutes later, 800 μL of sodium carbonate solution (75 g/L) was added, and the mixture was vortexed. The resulting solution was incubated in the dark at room temperature for 1 h, and the absorbance was measured at 765 nm. Total phenols were expressed in mg gallic acid equivalents (GAE)/g dry weight (dw), based on a standard curve prepared with known concentrations of gallic acid (0–200 mg/L).

#### Caffeine content

0.75 g of each sample was weighed in duplicates. HPLC-grade water was heated to 90°C on a hot plate, and then 50 mL was poured over each sample. The mixture was swirled lightly to homogenize, then left to brew on the bench for 15 min. Afterward, samples were filtered using Whatman 90-mm filter papers.

The filtered samples were diluted 1:5 in mobile phase (70% water and 30% methanol), then filtered using 0.2 μm nylon syringe filters [39].

Each sample was pipetted into an HPLC vial and injected in duplicate.

**Table:** 

Factor	Method
Column	C18 reversed phase 120 ODS-A, 5 mm (150 × 4 mm)
Temperature	Room temperature
Solvents	70 water:30 methanol
Gradient	No gradient
Flow	1 mL/min
Injection volume	10 μL
Duration	10 min
UV detection	254 nm

### Statistical analysis

All analyses were performed using GraphPad Prism (version 9.0.0) and R (version 4.5.0; R Core Team, R Foundation for Statistical Computing, Vienna, Austria), with significance set at *P* < 0.05. Data is presented as mean ± SD. Normality was assessed both for individual distributions with the Shapiro-Wilk test and for model residuals. The performed tests did not reject overall the normality of the considered variables or models when the observations are considered together. A mild deviation from normality is only observed for the variable delta efficiency (DE). Given the absence of major outliers, the normality assumption was considered reasonably met for the application of parametric statistical analysis.

Participants were grouped by BMI into normal weight (<25 kg/m^2^) and overweight/obese (≥25 kg/m^2^) categories. In our sample, 9 men and 10 women were of normal weight, whereas 6 men and 4 women fell within the overweight/obese category. Two-way repeated-measures analysis of variance (ANOVA) was used to examine the effects of drink (AYM, KYM, caffeine water, and plain water) and exercise intensity (20–80 W) on EE, respiratory exchange ratio (RER), HR, and DE. Sex and obesity status were included as between-subject factors. DE was calculated from the slope of EE—power linear regression [40].

A priori power analysis was conducted to assess the adequacy of the chosen sample size for detecting main effects and interactions in the repeated-measures ANOVA with a 4 Drinks × 4 Intensities design using the observed means, the estimated SD, and correlation estimates derived from the dataset. The analysis shows that the study presents, in general, a power of at least 90% to detect main effects of drinks across all outcomes. This indicates a strong statistical framework to understand the physiological changes caused by drinks. Similarly, an acceptable power is detected for the main effect of Intensity. Interaction terms, meaning Drink ∗ Intensity, were also evaluated, but with lower statistical power than the main effects of drinks and intensity. In particular, the power to detect interaction effects ranged from 60% to 80%, depending on the outcome variable and the assumed effect size. This supports the capacity to detect the variation of responses across intensities. Together, these results confirm the study design, these findings show that the sample size is sufficient to detect the effects of the different drinks on metabolic dynamics during low to moderate exercise.

## Results

### YM composition analysis

As shown in Table 1, the 2 brands of YM consumed by participants, AYM and KYM, differed slightly in their phytochemical composition. KYM shows higher total phenolic content than AYM (88.8 ± 3.7 GAE/g compared with 78.6 ± 2.4 GAE/g; *P* = 0.008). Both brands also contained substantial amounts of caffeine, with almost similar concentrations (AYM = 8.0 ± 0.3 mg/g compared KYM = 8.4 ± 1.2 mg/g; *P* > 0.05), corresponding to ∼59.7 mg and 62.7 mg per 500 mL infusion, respectively. These values align with previously published data on commercial YM products [41,42].

**TABLE 1:** Analyzed composition of yerba mate drink consumed by participants

	YM	SD	*P* value
**Total Phenols (GAE/g)**			
AYM	78.64	2.44	—
KYM	88.79	3.67	0.008∗
**Caffeine (mg/g)**			
AYM	7.96	0.26	—
KYM	8.36	1.18	0.63

GAE/g, milligrams of gallic acid equivalents per gram; YM, yerba mate; AYM, Amanda brand yerba mate; KYM, Kharta brand yerba mate.

### Participant characteristics

Participant characteristics are summarized in Table 2. The sample included 29 healthy young adults (15 men and 14 women), with a mean age of 21.3 ± 3.8 y. To compare men and women, independent samples t-tests have been performed when the normality and equality of variances assumptions were met; otherwise, the Mann-Whitney U-test was used. As expected, men were significantly heavier (*P* = 0.0055) and taller (*P* < 0.001) than women. Although BMI did not differ significantly between groups (*P* = 0.2385), fat mass percentage was significantly higher in women (32.3 ± 7.6%) compared with men (21.9 ± 13.0%, *P* = 0.0146), whereas fat-free mass was markedly higher in men (60.7 ± 6.6 kg compared with 40.3 ± 4.1 kg, *P* < 0.001).

**TABLE 2:** Characteristics of the study participants, presented as mean (± SD) for men (n = 15) and women (n = 14)

	Total (29)	Men (15)	Women (14)	*P* value
**Age (y)**	21.3 (3.8)	21.1 (4.2)	21.6 (3.5)	0.729
**Weight (kg)**	70.9 (19.4)	80.6 (20.4)	60.6 (11.9)	0.006∗
**Height (m)**	1.70 (0.09)	1.77 (0.07)	1.62 (0.04)	<0.001∗
**BMI (kg/m^2^)**	24.6 (6.2)	26.1 (7.4)	22.9 (4.2)	0.238
**Fat mass (%)**	26.9 (11.8)	21.9 (13.0)	32.3 (7.6)	0.015∗
**Fat mass (kg)**	20.1 (13.0)	19.9 (16.4)	20.3 (8.8)	0.305
**Fat-free mass (kg)**	50.9 (11.7)	60.7 (6.6)	40.3 (4.1)	<0.001∗
**Basal metabolic rate (kcal/min)**	1.421 (0.267)	1.566 (0.266)	1.265 (0.165)	0.001∗
**Fasting respiratory exchange ratio**	0.81 (0.06)	0.82 (0.06)	0.80 (0.06)	0.413
**Fasting heart rate (bpm)**	74 (8)	73(8)	76(7)	0.331

Values are mean (± SD), ∗*P* < 0.05.

Additionally, BMR was significantly higher in men (1.566 ± 0.266 kcal/min) than in women (1.265 ± 0.165 kcal/min, *P* = 0.0012), reflecting differences in fat-free mass. No significant sex differences were observed in fasting RER or resting HR (*P* > 0.05).

### Metabolic changes to YM during exercise

**FIGURE 3:**
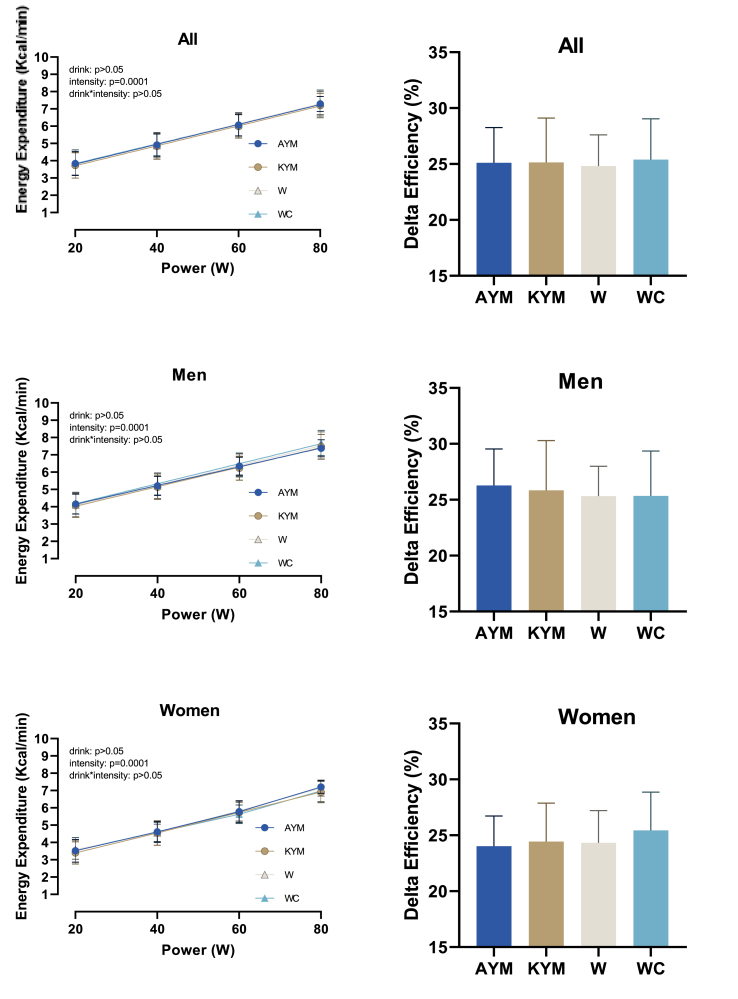
Effect of Drink (AYM, KYM, W, and WC) on energy expenditure and delta efficiency across different intensities (20W, 40W, 60W, and 80W) in 29 participants (15 men and 14 women). YM, yerba mate; AYM, Amanda brand yerba mate; KYM, Kharta brand yerba mate; W, ater; WAC, water and caffeine.

All beverages (AYM, KYM, water, and caffeinated water) were evaluated for their effects on EE, RER, and HR during progressive submaximal cycling (20–80 W). According to the 2-way repeated-measures ANOVA, EE significantly increased with exercise intensity (*P* < 0.001, partial η^2^ = 0.990), regardless of the beverage consumed (*P* = 0.723), as shown in Figure 3 and Table 3. This confirms that workload is the dominant factor influencing EE.

**FIGURE 4:**
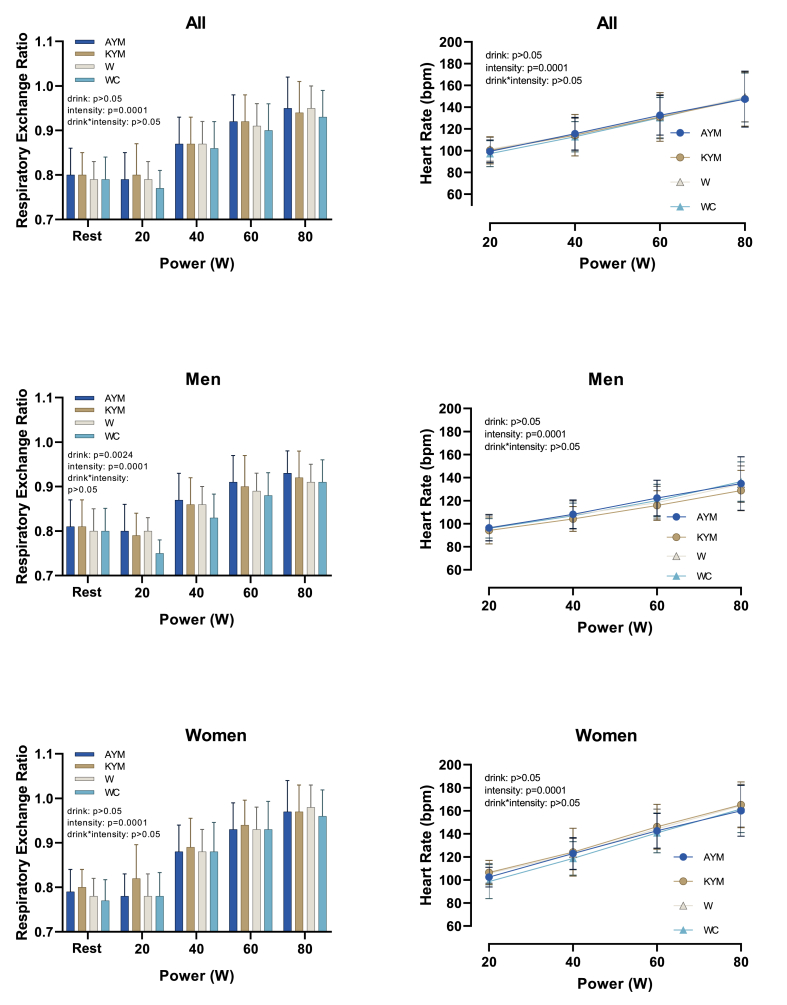
Effect of the drink (AYM, KYM, W, and WC) on respiratory exchange ratio and HR across different intensities in 29 participants (15 men and 14 women). YM, yerba mate; AYM, Amanda brand yerba mate; KYM, Kharta brand yerba mate; W, ater; WAC, water and caffeine.

Similarly, RER values rose with intensity (*P* < 0.001, partial η^2^ = 0.916), reflecting a physiological shift from fat to carbohydrate metabolism (Figure 4).

HR followed a similar pattern, increasing with exercise intensity (*P* < 0.001, partial η^2^ = 0.922), but was unaffected by beverage type (*P* = 0.942) or any of its interactions.

No significant main effects of beverage type were found for EE, RER, or HR (all *P* > 0.05), suggesting that neither YM brand nor caffeine-enriched water altered metabolic or cardiovascular responses during exercise.

From the within-subjects analysis shown in Table 4, DE did not significantly differ across beverage types or participant sex (*P* = 0.595), as also shown in Figure 3. This indicates that acute YM ingestion, regardless of brand, did not enhance exercise efficiency under these conditions.

### Sex-based differences

Sex-related effects were primarily observed in substrate utilization (Figure 4) and cardiovascular responses. Specifically, based on Table 3, the interaction between intensity and sex appeared to be significant for RER (*P* = 0.002, partial η^2^ = 0.225) and for HR (*P* = 0.001, partial η^2^ = 0.289). These findings indicate that women exhibited significantly higher RER and HR values at increasing exercise intensities compared with men, suggesting a greater reliance on carbohydrate metabolism and consistent with known differences in cardiovascular regulation between sexes. This pattern aligns with the understanding that glucose oxidation is more oxygen-efficient than fatty acid oxidation and may therefore support higher cardiac efficiency during graded exercise [43].

**TABLE 3:** Within-subject effects tests: Interaction effects of obesity status, sex, drink, and intensity on cardiometabolic variables

	EE					RER					HR				
	Sum of squares	DF	F ratio	Sig.	Effect size	Sum of squares	DF	F ratio	Sig.	Effect size	Sum of squares	DF	F ratio	Sig.	Effect size
					(partial *η*^2^)					(partial η^2^)					(partial η^2^)
**Drink**	0.515	2.285	0.365	0.723	0.014	0.022	2.678	1.547	0.214	0.058	66.942	2.359	0.085	0.942	0.003
**Drink ∗ Sex**	0.842	2.285	0.598	0.575	0.023	0.024	2.678	1.657	0.189	0.062	655.616	2.359	0.831	0.458	0.032
**Drink ∗ Obesity status**	0.610	2.285	0.433	0.677	0.017	0.024	2.678	1.726	0.175	0.065	46.180	2.359	0.059	0.962	0.002
**Drink ∗ Sex ∗ Obesity status**	0.634	2.285	0.450	0.666	0.018	0.027	2.678	1.885	0.147	0.070	1396.242	2.359	1.770	0.173	0.066
**Intensity**	633.620	2.368	2475.869	0.000	0.990	1.358	1.923	271.025	0.000	0.916	112291.095	1.367	294.930	0.000	0.922
**Intensity ∗ Sex**	0.329	2.368	1.286	0.286	0.049	0.036	1.923	7.244	0.002	0.225	3859.828	1.367	10.138	0.001	0.289
**Intensity ∗ Obesity status**	1.607	2.368	6.280	0.002	0.201	0.006	1.923	1.223	0.302	0.047	3178.673	1.367	8.349	0.003	0.250
**Intensity ∗ Sex ∗ Obesity status**	0.183	2.368	0.717	0.515	0.028	0.011	1.923	2.167	0.127	0.080	64.531	1.367	0.169	0.760	0.007
**Drink ∗ Intensity**	0.316	4.587	0.636	0.659	0.025	0.003	5.186	0.405	0.851	0.016	222.953	4.173	0.853	0.499	0.033
**Drink ∗ Intensity ∗ Sex**	0.763	4.587	1.537	0.189	0.058	0.015	5.186	2.265	0.050	0.083	166.146	4.173	0.635	0.645	0.025
**Drink ∗ Intensity ∗ Obesity status**	0.272	4.587	0.549	0.724	0.021	0.004	5.186	0.656	0.663	0.026	108.651	4.173	0.415	0.805	0.016
**Drink ∗ Intensity ∗ Sex ∗ Obesity status**	0.925	4.587	1.862	0.113	0.069	0.003	5.186	0.429	0.834	0.017	181.283	4.173	0.693	0.604	0.027

EE, energy expenditure; RER, substrate oxidation; HR, heart rate; Sig, significance, DF, degrees of freedom.

Additionally, no standard drink × sex interaction was observed for within-subject ANOVA test effects’ results for HR, RER, and EE (all *P* > 0.05), indicating that sex did not influence how different beverages affected these variables.

However, when intensity was added to the equation (drink × intensity × sex), significance appeared, indicating that drink type affects EE differently by sex and exercise intensity (Table 5) and more specifically in the RER data of men (Figure 4).

**TABLE 4:** Within-subject effects tests: interaction effects of obesity status, sex, and drink on delta efficiency

DE					
	Sum of squares	DF	F ratio	Sig.	Effect size
					(partial *η*^2^)
**Drink**	16.430	3	0.616	0.595	0.024
**Drink ∗ Sex**	41.617	3	1.560	0.210	0.059
**Drink ∗ Obesity status**	4.251	3	0.159	0.912	0.006
**Drink ∗ Sex ∗ Obesity status**	65.488	3	2.455	0.070	0.089

DE, delta efficiency; DF, degrees of freedom.

**TABLE 5:** Within-subject contrast tests: effects of drink, sex, obesity status, and intensity on energy

	EE					RER					HR				
	**Sum of squares**	**DF**	**F Ratio**	**Sig.**	**Effect size**	**Sum of squares**	**DF**	**F Ratio**	**Sig.**	**Effect size**	**Sum of squares**	**DF**	**F Ratio**	**Sig.**	**Effect size**
					**(partial η^2^)**					**(partial η^2^)**					**(partial η^2^)**
**Drink**	0.210	1.000	0.447	0.510	0.018	0.000	1.000	0.013	0.910	0.001	59.910	1.000	0.239	0.629	0.009
**Drink ∗ Sex**	0.305	1.000	0.648	0.428	0.025	0.019	1.000	5.953	0.022	0.192	440.549	1.000	1.759	0.197	0.066
**Drink ∗ Obese**	0.021	1.000	0.044	0.836	0.002	0.000	1.000	0.153	0.699	0.006	1.392	1.000	0.006	0.941	0.000
**Drink ∗ Sex ∗ Obese**	0.529	1.000	1.124	0.299	0.043	0.023	1.000	7.072	0.013	0.220	212.663	1.000	0.849	0.366	0.033
**Intensity**	0.603	1.000	8.710	0.007	0.258	0.054	1.000	51.609	0.000	0.674	120.009	1.000	3.093	0.091	0.110
**Intensity ∗ Sex**	0.109	1.000	1.576	0.221	0.059	0.000	1.000	0.000	0.995	0.000	34.468	1.000	0.888	0.355	0.034
**Intensity ∗ Obese**	0.076	1.000	1.092	0.306	0.042	0.004	1.000	3.742	0.064	0.130	4.130	1.000	0.106	0.747	0.004
**Intensity ∗ Sex ∗ Obese**	0.001	1.000	0.010	0.922	0.000	0.000	1.000	0.143	0.708	0.006	0.549	1.000	0.014	0.906	0.001
**Drink ∗ Intensity**	0.010	1.000	0.237	0.631	0.009	0.000	1.000	0.013	0.910	0.001	23.538	1.000	1.714	0.202	0.064
**Drink ∗ Intensity ∗ Sex**	0.185	1.000	4.480	0.044	0.152	0.000	1.000	0.163	0.690	0.006	0.079	1.000	0.006	0.940	0.000
**Drink ∗ Intensity ∗ Obese**	0.052	1.000	1.258	0.273	0.048	0.001	1.000	0.741	0.398	0.029	16.552	1.000	1.205	0.283	0.046
**Drink ∗ Intensity ∗ Sex ∗ Obese**	0.070	1.000	1.683	0.206	0.063	0.000	1.000	0.053	0.820	0.002	2.833	1.000	0.206	0.654	0.008

EE, energy expenditure; RER, substrate oxidation; HR, heart rate; DF, degrees of freedom.

### Influence of obesity status

Obesity status (normal compared with overweight/obese) significantly influenced several exercise parameters. For instance, according to Table 3, the interaction Intensity × Obesity status appeared to be significant for EE (*P* = 0.002, partial η^2^ = 0.201), and HR (*P* = 0.003, partial η^2^ = 0.250). These results suggest that individuals with higher obesity show altered EE and HR responses to increasing workload compared with their nonobese counterparts. This could potentially be due to higher metabolic cost or cardiovascular pressure [44,45]. The main effect of obesity and intensity on RER was not statistically significant (*P* = 0.302). However, according to Table 4 of within-subject contrast tests, a borderline trend toward significance was noted for RER by the interaction Intensity × Obesity status (*P* = 0.064), suggesting subtle differences in substrate metabolism that merit further investigation. However, no significant beverage × obesity interactions were found for any metabolic or cardiovascular outcome. Beverage type did not differentially affect obese compared with nonobese participants.

A significant 3-way interaction between drink, sex, and obesity status (Dring × sex × Obesity status) was observed for RER (*P* = 0.013, partial η^2^ = 0.220), although the overall effect size was moderate (Table 5).

Interestingly, a trend level 3-way interaction between drink, sex, and obesity status appears (*P* = 0.070, partial η^2^ = 0.089) but with a small effect size (Table 4), suggesting a possible combined influence of participant characteristics on DE.

## Discussion

This study aimed to investigate the acute effects of 2 commercially available brands of brewed YM on metabolic and cardiovascular responses during very low to moderate-intensity cycling comparable with workloads typical of light daily activities. The findings indicate that, despite differences in polyphenol content between the brands, YM consumption did not significantly influence EE, substrate oxidation, HR, or DE. The small magnitude of the observed changes suggests limited clinical relevance, as the responses fell within normal physiological variability and are unlikely to translate into meaningful metabolic or performance benefits.

### YM composition

Compositional analysis revealed that KYM had higher polyphenol content, whereas no differences in caffeine between brands. The reported differences are consistent with previous studies reporting variability in YM composition due to processing and leaf selection methods [46]. Both brands were within the typical caffeine range for YM products [13].

Despite the differences in phenolic content, no significant effects were observed on EE, RER, or HR. One plausible explanation is that the caffeine dose provided (∼135 mg in the WC condition) may have been too modest to produce a detectable thermogenic response under the controlled, short-duration conditions of this study. Additionally, polyphenol bioavailability is known to be limited, particularly in acute settings, and its thermogenic synergy with caffeine may not be sufficient unless administered chronically [47,48]. The fact that beverages were served at room temperature may also have reduced potential thermogenic effects, as cold beverage ingestion has been shown to amplify EE [49].

### Participant characteristics and substrate metabolism

Sex-based differences in anthropometric characteristics aligned with established norms, with men exhibiting higher fat-free mass and BMR, whereas women presented higher body fat percentage [50,51]. These differences in body composition are key physiological determinants of resting EE and substrate utilization, as fat-free mass primarily drives metabolic rate, whereas adiposity influences patterns of lipid and carbohydrate oxidation [52,53]. No significant difference in fasting RER was observed between sexes, consistent with previous findings that resting metabolic patterns are similar across sexes when adjusted for lean mass [54].

#### Metabolic changes to YM ingestion during exercise

Preclinical research has consistently demonstrated that YM enhances lipid metabolism and promotes thermogenesis [55,56]. However, such effects are generally observed with chronic supplementation and may not be evident after a single, acute dose, as in the current study. Nonetheless, human trials in endurance athletes show that a single YM drink, or a few days of repeated intake, can acutely increase fat oxidation, lower respiratory exchange ratio, and modestly improve cycling time-trial performance when consumed 40 to 60 min before exercise [23,26].

One possible explanation lies in the quantity and bioavailability of the active compounds. Previous research has shown that a caffeine dose of 200 to 300 mg is generally required to elicit a significant increase in metabolic rate [47]. In our study, the caffeine content of the YM drinks may have been too low to exceed this threshold, especially when distributed across body weight, as smaller individuals receive a higher caffeine dose per kilogram [57]. Importantly, caffeine reaches peak plasma concentrations ∼30 to 60 min after ingestion, depending on beverage form and gastric emptying, suggesting that bioavailability and timing relative to exercise onset may have influenced the observed metabolic responses during submaximal cycling [16]. Moreover, although YM is rich in polyphenols, their bioavailability is limited, and their thermogenic effect is often dependent on their interaction with caffeine [48,58]. Boado et al. [59] determined bioavailability of polyphenols in YM, which was found to be ∼49.3% over a 120-min period. It is also worth noting that studies conducted over 13.5 to 24 h in respiratory chambers have generally shown a stronger trend, though not always statistically significant, toward a lower RER, indicating increased fat oxidation, when participants consumed green tea products containing both caffeine and catechins compared with caffeine alone [25]. Taken together, these acute YM and tea-based studies suggest that meaningful short-term changes in EE and substrate use are biologically plausible, but highly sensitive to factors such as caffeine/polyphenol dose, pre-exercise nutritional status, exercise intensity, and training level [23], factors that may have collectively limited the observable impact of our single, commercially brewed YM dose during low- to moderate-intensity cycling.

Processing methods may also play a role. Heat treatments such as drying or roasting can degrade polyphenols and other thermolabile compounds, potentially reducing the functional or thermogenic impact of YM [46,60]. As such, even though YM is known to have higher antioxidant potential than green or black tea [55], its metabolic effects may not always translate into acute physiological responses during exercise.

### Sex-based differences

During exercise, women exhibited significantly higher RER values at moderate and high intensities, suggesting greater reliance on carbohydrate metabolism. This aligns with prior studies showing that sex hormones and muscle fiber composition can influence substrate use during exercise [54,61].

Women also showed significantly higher HR at the same workloads, consistent with known cardiovascular differences, such as smaller heart size and reduced stroke volume [62,63].

Interestingly, although sex influenced overall physiological responses to exercise intensity, it did not significantly affect the acute impact of beverage type on RER, HR, or EE (*P* > 0.05). This suggests that the ergogenic or metabolic effects of the drinks were generally comparable across sexes. However, a significant 3-way interaction (drink × intensity × sex) was observed, particularly impacting RER values in men, indicating that drink type may differentially influence metabolic responses depending on both sex and exercise intensity.

### Influence of obesity status

Obesity status significantly influenced EE and HR during exercise. Participants with higher adiposity displayed steeper increases in both metrics with rising workload, likely due to greater energy demands and cardiovascular strain during physical activity [64]. A borderline trend in RER (*P* = 0.064) hints at altered substrate use in individuals with obesity, possibly due to impaired mitochondrial function or hormonal dysregulation [51,65]. Though drink × obesity interactions were not significant, these results suggest that obesity may modify physiological responses to exercise more than acute beverage ingestion.

Recent research by Fares et al. [66] found that in healthy young adults, body fat, particularly trunk and limb fat, was positively correlated with DE in women, but not in men. These findings may imply that fat distribution may affect mechanical efficiency in women during submaximal exercise. This may reflect biomechanical adaptations or energy conservation mechanisms that emerge with higher segmental adiposity.

### DE interactions

Analysis of DE revealed no significant main effects of beverage type on exercise efficiency across workloads. Although mean DE values varied slightly between treatments, these differences did not reach statistical significance. As shown in Table 4, there were no meaningful interactions between treatment conditions and workload for DE. This suggests that, under the tested conditions, acute ingestion of YM, regardless of brand, did not alter mechanical efficiency during submaximal cycling.

### Limitations

This study was limited by the relatively low content of both caffeine and polyphenols in the brewed YM, which may have been insufficient to elicit thermogenic or ergogenic effects. As the intervention was acute, it may not capture the benefits associated with longer-term YM intake. The findings remain specific to young, healthy adults and may not generalize to other populations. Future research should examine higher doses, extended supplementation, and varied exercise modalities (based on relative intensities) to better understand YM’s potential metabolic and ergogenic effects.

In conclusion, acute ingestion of commercially brewed YM, despite its caffeine and polyphenol content, did not significantly alter EE, substrate oxidation, HR, or exercise efficiency during very low- to moderate-intensity cycling. These results suggest that body composition may have a more profound impact on physiological responses to exercise than short-term nutritional interventions, such as brewed YM consumption. Future research should explore the long-term effects of YM, evaluate higher doses of active compounds, and assess segmental body composition outcomes across diverse exercise intensities and modalities. It will also be important to ensure that comparison treatments are matched for equivalent bioactive concentrations to allow clearer interpretation of effects.

## Author contributions

The authors’ responsibilities were as follows – EJF: conceptualization; EJF: design of the work; SZ, MZ, RZE: acquisition of data; EJF, RB, SZ: data analysis; EJF, RE: interpretation of data; EJF: drafting the work; IT, OO: substantive revision of the work. All authors read and approved the final manuscript.

## Ethics approval and consent to participate

The study was conducted in accordance with the guidelines of the Declaration of Helsinki and approved by the Institutional Review Board of the American University of Beirut (protocol code BIO-2022-0200 - November 27, 2023). All participants voluntarily participated in this study and provided written informed consent.

## Data availability

All data generated or analyzed during this study are included in this published article.

## Funding

This study was funded by the American University of Beirut Research Board (project #: 25827, award #: 104110).

## Conflict of interest

The authors declare that they have no competing interests.
