# Supplementary material for: Acute Effects of Commercial Yerba Mate Products on Cardiometabolic Responses during Submaximal Cycling: Brewed to Perform?
Source: Curr Dev Nutr. 2026 Jan 16;10(2):107637. doi: 10.1016/j.cdnut.2026.107637 (PMC12906188; doi:10.1016/j.cdnut.2026.107637)
Supplement: Multimedia component 1 [file mmc1.docx]

****Additional File 1:**

File Format: .xls

Title of Data: Interaction effects of different parameters on cardiometabolic variables.

Description of data: This file contains the Interaction effects of obesity status, sex, drink, and intensity on energy expenditure (EE), delta efficiency (DE), substrate oxidation (RER), and heart rate (HR) trends.

****Additional File 2:**

File Format: .xls

Title of Data: effects of beverages on cardiometabolic variables.

Description of data: This file contains the effects of beverages (AYM, KYM, water, and caffeinated water) on EE, RER, and HR during progressive submaximal cycling (20–80 W) in men and women.
